# Giant defect emission enhancement from ZnO nanowires through desulfurization process

**DOI:** 10.1038/s41598-020-61189-7

**Published:** 2020-03-06

**Authors:** Junze Zhou, Komla Nomenyo, Clotaire Chevalier Cesar, Alain Lusson, Adam Schwartzberg, Chun-Chieh Yen, Wei-Yen Woon, Gilles Lerondel

**Affiliations:** 1 Lumière, Nanomatériaux, Nanotechnologies, CNRS ERL 7004, Institut Charles Delaunay, Université de Technologie de Troyes, 12 Rue Marie Curie, CS 42060, 10004 Troyes, Cedex France; 2 Groupe d’Etude de la Matière Condensée, Université de Versailles, Saint Quentin-en-Yveline, 45 Avenue de Etat Unis, 78035 Versailles, France; 3 Molecular Foundry, Lawrence Berkeley National Laboratory, 1 Cyclotron Road, Berkeley, California 94720 USA; 4 Department of Physics, National Central University, Taoyuan, 32001 Taiwan, R.O.C.

**Keywords:** Materials science, Materials science, Materials for optics, Materials for optics

## Abstract

Zinc oxide (ZnO) is a stable, direct bandgap semiconductor emitting in the UV with a multitude of technical applications. It is well known that ZnO emission can be shifted into the green for visible light applications through the introduction of defects. However, generating consistent and efficient green emission through this process is challenging, particularly given that the chemical or atomic origin of the green emission in ZnO is still under debate. In this work we present a new method, for which we coin term desulfurization, for creating green emitting ZnO with significantly enhanced quantum efficiency. Solution grown ZnO nanowires are partially converted to ZnS, then desulfurized back to ZnO, resulting in a highly controlled concentration of oxygen defects as determined by X-ray photoelectron spectroscopy and electron paramagnetic resonance. Using this controlled placement of oxygen vacancies we observe a greater than 40-fold enhancement of integrated emission intensity and explore the nature of this enhancement through low temperature photoluminescence experiments.

## Introduction

Wurtzite ZnO is a promising semiconductor with a large direct band-gap (~3.37 eV) and high exciton binding energy (~60 meV) at room temperature. Thin films comprised of ZnO nanowires are of particular interest for optoelectronic applications such as nanolasers^1^, gas sensors^2^, light emitting diodes^3^ and photocatalysis^4^. Photoluminescence (PL) spectra of ZnO nanowires^5–8^ are generally comprised of two main features^9^: near band-gap ultraviolet (UV) emission and a broad visible emission ranging from green to red, which is related to the concentration of point and structural defects in the material. The ability to generate this broad visible emission is of significant interest for phosphors. Through careful control of growth processes and post-growth modifications it is possible to control intensity and spectral shape making these materials a near-ideal, elementally abundant, replacement for rare-earth reliant phosphors.

Nanowires synthesized by solution growth have a smooth, wide PL peak centered at ~2 eV^10–12^ due to zinc vacancy complexes^10^. Vapor grown nanowires^13–15^ have their PL centered in the green, dubbed the green band, and commonly attributed to oxygen vacancies^16–18^, zinc vacancies^19^ or interstitials^20^. More recently, the defect structure investigations of ZnO by PL have been complemented by the aid of electron paramagnetic resonance (EPR) spectroscopy^20–24^ and a core-shell model has been introduced for distinguishing the surface and core defects emission. Vacuum annealing at 900 °C^25^ is an effective post growth synthetic modification that produces oxygen deficient ZnO:Zn with enhanced green/white emission^26^. Chemical changes can also be used to induce significant control over PL properties; Simmons *et al*.^27^ recently demonstrated that the sulfidation of ZnO powders can produce a greater than two-fold enhancement in defect emission.

In this paper, we describe a simple route to create a ZnO nanowire thin film via post growth sulfurization/desulfurization with significantly enhanced defect emission. The modified ZnO thin film exhibits significant green emission enhancement as compared with the as grown sample with a strong correlation between oxygen vacancies and green emission. Temperature dependent PL spectroscopy shows a dominant excitonic feature at ~3.24 eV at low temperature, which is related to phonon replicas of both a defect related transition and the free exciton. The green emission at ~2.5 eV presents a negative thermal quenching (NTQ) with a thermal activation energy of about 5.3 meV which shows a good agreement to the ambient thermal energy at 70 K. Traditionally, ZnO:Zn with high defect emission can be created directly through the use of very high temperature processes. We have shown here that through the sulfurization/desulfurization process strong defect emission can be induced at lower temperatures with greater control over degree of defects induced. This is an important advancement, particularly in the field of earth-abundant phosphors.

## Experimental Details and Analysis Method

ZnO nanowires were prepared by chemical bath deposition on quartz and silicon substrates. 0.035 M Zinc acetate was dissolved in 750 mL of DI water. 9 ml of ammonium hydroxide was added to this solution. The ZnO seed layer pre-coated quartz and silicon substrates were immersed in this solution for one hour at 87 °C (details of the deposition technique have been reported elsewhere^28^). The desulfurized ZnO was prepared via two steps, namely sulfurization and desulfurization, in a chemical vapor deposition (CVD) reactor (a horizontal tube furnace). The CVD furnace used in the experiment has been reported previously^29^. For the sulfurization process, the ZnO nanowire sample was exposed to an H_2_S flow of 2 sccm and an Ar flow of 100 sccm at 550 °C for 10 min. For the desulfurization process, the sulfurized ZnO sample was treated in air at 550 °C for 60 min, in order to thermally extract the sulfur.

Crystal structures were measured by X-ray diffraction (INEL EQUINOX100) with Cu Kα_1_ radiation (**λ** = 1.5406 Å, at 30 kV and 0.9 mA). A HITACHI S-3400N scanning electron microscope was used to study the morphology of the samples. The PL spectra were acquired using a He-Cd laser (325 nm) as an excitation source, and an ANDOR SR500i spectrometer equipped with a CCD camera. The optical transmission curves of the samples were measured using a VIS-NIR spectrophotometer (Cary 100), with a spot size of about 2 mm × 5 mm. Low temperature PL spectroscopy was conducted in a cryostat cooled by liquid helium. X-ray photoelectron spectroscopy (XPS) was measured by a high-resolution ULVAC-PHI XPS: PHI Quantera SXM. EPR was conducted at 10 K. The sample was placed in a standard 4102ST cavity. The typical EPR instrumental settings were: center field 3455 G, microwave frequency 9.47 GHz, microwave power 15 mw, sweep width 100 G, modulation frequency 100 kHz, modulation amplitude 1.6 G, receiver gain 30, and time constant 32.88 ms. The oxygen plasma treatment was carried out in a reactive ion etcher (PLASSYS MU400). The desulfurized samples was exposed to a plasma comprised of 10 sccm oxygen at 20 mTorr for 5 min. To estimate the volume fraction of ZnS and ZnO in the sulfurized sample, light transmission through the as grown sample and the sulfurized one were simulated based on the transfer matrix method^30^. A rough porous thin film model was applied to fit the transmission curves. Based on the assumption of a small angle scattering, rough Fresnel coefficient^31^ was applied in the calculation matrix^32^. As for the modeling on the porous properties of the thin film, we selected the Landau Lifshitz/Looyenga model^33,34^ as the effective medium model. The effective permittivity can be deduced from following equation:

1 $$\sqrt[3]{{\varepsilon }_{eff}}={V}_{1}^{3}\sqrt{{\varepsilon }_{1}}+{V}_{2}^{3}\sqrt{{\varepsilon }_{2}}+\cdots +{V}_{n}^{3}\sqrt{{\varepsilon }_{n}}$$

while *V*_*n*_ and *ε*_*n*_ are the volume fraction and permittivity of the component *n*.

## Results and Discussion

The traditional method of preparing ZnO:Zn involves maintaining ZnO powder at a high temperature (~900 °C)^24^. In our two-step vapor-based method, we use sulfur as an intermedium to effectively generate defects by treating the sulfurized ZnO nanowires in air at temperatures as low as 550 °C to remove the sulfur. The characterization results are presented in two parts: sulfurization and desulfurization.

**Figure 1:**
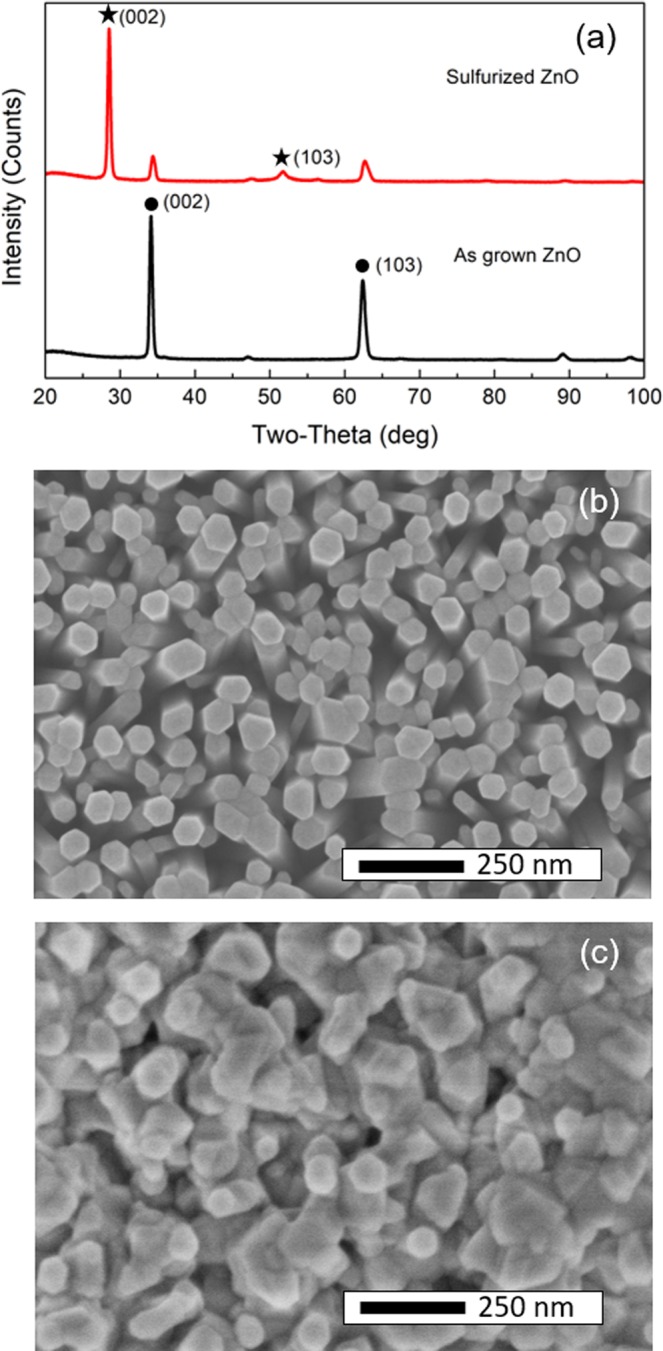
Sulfurization characterization: (**a**) XRD patterns of the as grown ZnO and sulfurized ZnO. The point symbols denote the (002) and (103) crystalline planes of the hexagonal structure of ZnO and the star symbols for that of ZnS. (**b**) The SEM images of as grown ZnO and sulfurized ZnO taken from the top and cross-section (inset) sides, the scale bars are 250 nm.

Figure 1(a) shows XRD patterns of the as grown and sulfurized ZnO samples, which reveal both samples have (002) and (103) planes of unstrained wurtzitic ZnO at around 34.1° and 62.4°. In addition to the ZnO peaks, the sulfurized sample shows two additional diffraction peaks at 28.52° and 51.82°, which closely match with the (002) and (103) crystalline planes of the wurtzitic ZnS in the Crystallography open database CIF1101051. Both ZnS and ZnO are wurtzitic structure, which is a good indication that the ZnS is coming form the replacement of Sulfur atom by the Oxygen atom. For wurtzitic ZnS, the lattice constant along the *c* axis is 6.62 Å, which is larger than that of wurtzitic ZnO (c = 5.2 Å). Figure 1(b,c) show the morphologies of the as grown and sulfurized ZnO nanowires. In both top and cross-section views it is clear that the sulfurization process has expanded the volume of the as grown ZnO. As the ZnS has a larger lattice constant, the sulfurized sample has a bigger average diameter and thus less space between the nanowires as compared with the as grown sample.

**Figure 2:**
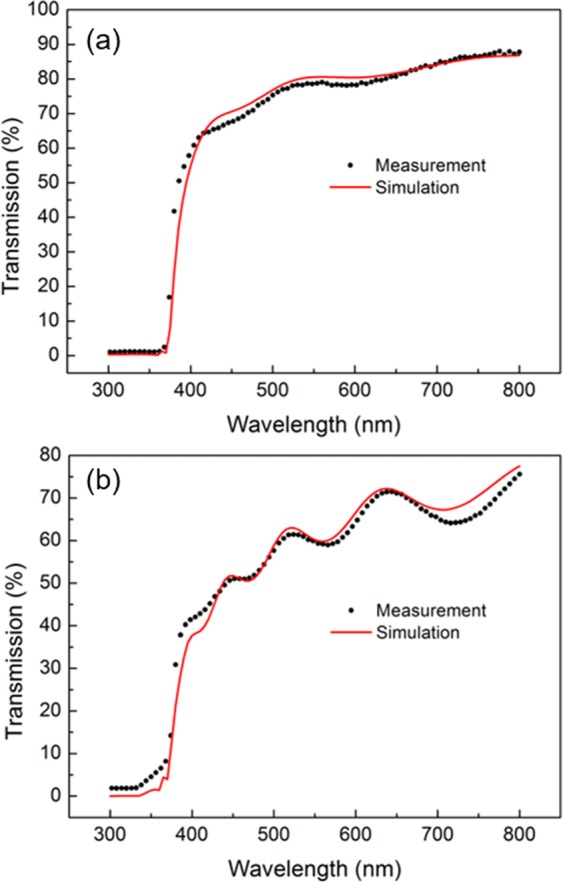
Porosity and proportion assessment: Transmission analysis of the as grown sample (**a**) and the sulfurized sample. (**b**) For both images, black dots and red line correspond to the experimental and stimulated curves respectively.

The porous thin film model was applied to estimate the volume proportion of each component in the as-grown and sulfurized samples. For the as-grown ZnO nanowire thin film the simulated curve in Fig. 2(a) is calculated based on the following parameters: the thickness of ZnO is 450 nm, the roughness amplitude is 25 nm, and the volume fraction of ZnO is 70%. This optimized curve fit well with the measured curve, while the thickness is known from cross sectional SEM, and we found the volume fraction is fairly comparable to the density of ZnO nanowires. As for the sulfurized ZnO, by optimizing the fitting (cf. Figure 2(b)), we found that the volume fraction of ZnS, ZnO and Air is about 56%, 19% and 25% respectively. The thickness is about 660 nm, which is comparable to the measured value as well. The transmission calculation indicates that ZnO is quite sensitive to the H_2_S gas; after sulfurization a significant proportion of the ZnO has been converted to ZnS.

**Figure 3:**
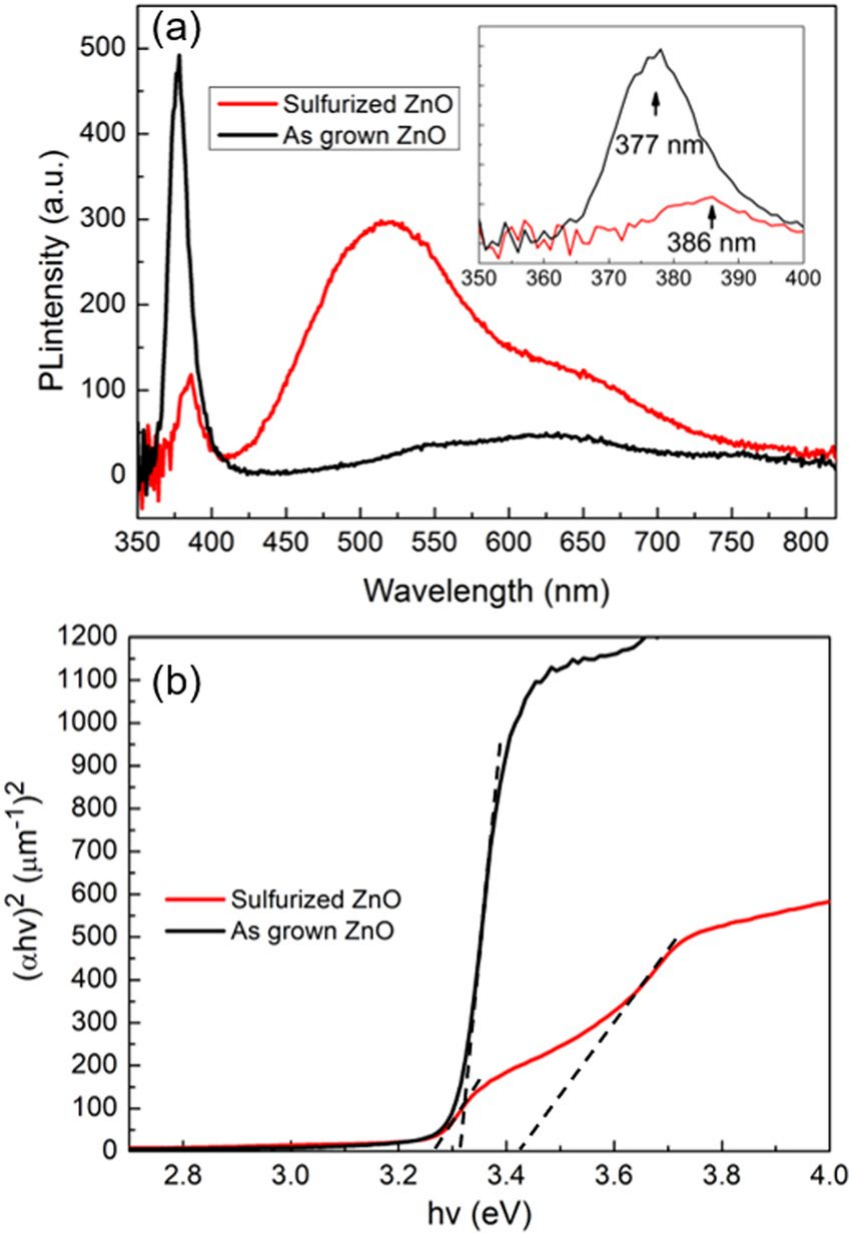
PL spectra and bandgap absorption of the sample before and after the sulfurization: (**a**) Room temperature PL spectra of the as grown and sulfurized sample. The inset represents the UV emission of the two samples, the y axis is the same as the one for the PL spectra. (**b**) Tauc plot of the as grown and sulfurized samples. Clearly two slope changes can be observed in the sulfurized sample, which are induced by the existence of two phases composed of ZnO and ZnS. The dot lines are a guide to the eyes for the different bandgap positions.

Room temperature PL was then conducted to observe optical properties of the sulfurized ZnO. As shown in the Fig. 3(a), the UV emission of the sulfurized sample decreases significantly and shifts to the red, as shown in the inset. This effect has been observed by Y. Yoo *et al*.^35,36^ and is caused by the size difference between S and O atoms. The redshift is further evaluated by the Tauc plot (cf. Figure 3(b)), while the sulfurized ZnO has another obvious bandgap feature at ~3.4 eV which corresponds to the bandgap transition of ZnS. Additionally, the sulfurized ZnO sample show significantly enhanced green emission (~510 nm) over the as-grown sample. The integrated intensity in the visible light emission is enhanced by 5-fold, while the ratio between defect and UV emission is enhanced by ~40 times, significantly higher than the value reported by G. Shen *et al*.^37^. The mechanism of this enhancement has recently been proposed in the report of Jay G. Simmons *et al*. The ZnS shell is formed around the ZnO core which traps photo-excited holes from the valence band. More electrons in the conduction band become available for deep level transitions, which results in the enhanced defect emission^27^.

**Figure 4:**
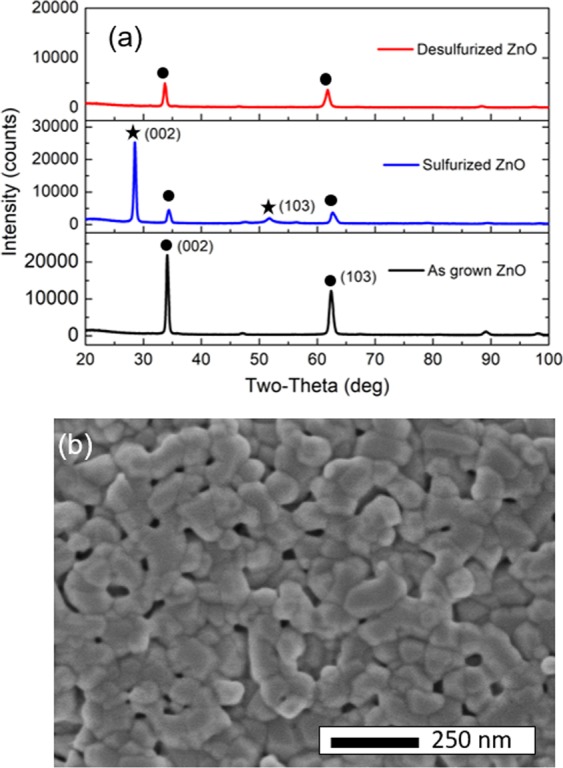
Desulfurization characterization: (**a**) XRD patterns of the as grown ZnO, sulfurized ZnO and the desulfurized sample. The point and star symbols denote the (002) and (103) crystalline planes of the hexagonal structure of ZnO and ZnS. (**b**) SEM image of the desulfurized sample, the inset picture is the cross-section view, the scale bars are 250 nm.

Figure 4(a) shows XRD patterns of the desulfurized, as grown and sulfurized samples. The desulfurized ZnO does not exhibit the specific peaks of ZnS, which indicates that the ZnS has been completely converted back to ZnO after the oxidation treatment. As shown in the Fig. 4(b) the sulfurized and desulfurized ZnO have similar porosity, and the desulfurized ZnO is less porous than as-grown. This suggests that the sulfurization process is not completely reversible, resulting in a lower density ZnO with a high concentration of defects

**Figure 5:**
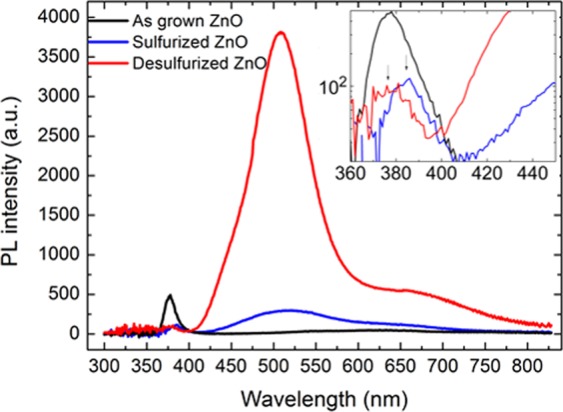
Room temperature PL spectra of the as grown, sulfurized and desulfurized ZnO. The inserted figure is the PL spectra represented in logarithmic scale.

Figure 5 shows the PL spectra of the desulfurized ZnO in direct comparison to the as-grown and desulfurized samples. The green defect emission at ~510 nm is significantly enhanced as compared with the as-grown sample, while the UV emission is almost complete quenched. The inset shows that the UV emission has shifted back to the same position as the as-grown sample, which may be due to the elimination of strain by the desulfurization. The integrated defect emission from 410 nm to 829 nm is enhanced by 5 times as compared with the emission of the sulfurized sample, while the total emission is enhanced by ~42 times compared with the emission of the as grown sample. To our knowledge, this ratio is the highest that has ever been reported, which indicates very efficient energy transfer from UV to visible emission.

**Figure 6:**
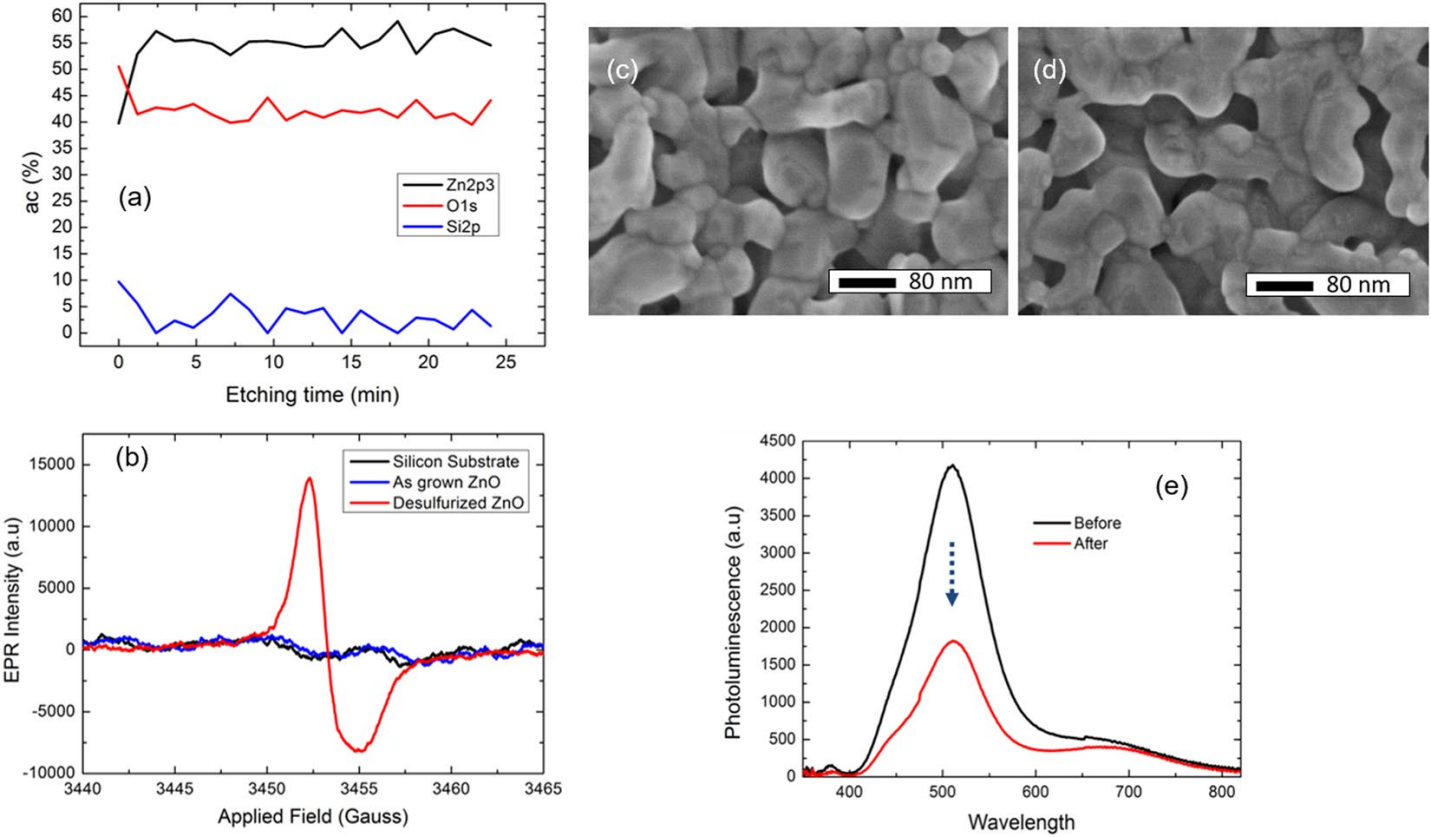
Defect characterizations of the desulfurized ZnO: (**a**) XPS profile as a function of the etching time, the etching speed was 8.3 nm/min. (**b**) Low temperature EPR of the silicon substrate, as grown ZnO and the desulfurized ZnO. SEM image of the desulfurized ZnO before (**c**) and after (**d**) the oxygen plasma treatment. (**e**) Room temperature PL spectra of the sample before and after the oxygen plasma treatment.

To investigate the origin of the green emission, we have examined the atomic ratio of the desulfurized ZnO by XPS. As shown in the Fig. 6(a), the zinc to oxygen ratio is approximately 6:4 for the desulfurized sample, which indicates that this sample is significantly oxygen deficient. Figure 6(b) shows low temperature EPR spectra. As compared with the case of the silicon substrate and as-grown ZnO sample, only the desulfurized ZnO exhibits a resonance at g-factor ~ 1.9678, which has been assigned to oxygen vacancies^27^. The lack of resonance for the as grown ZnO is probably due to the detection limit of the instrument. Nevertheless, the above EPR measurement indicates that the desulfurization processes did result in more abundant oxygen vacancies. We have also explored the possibility of passivating the green emission by the oxygen plasma treatment. The desulfurized ZnO was exposed to oxygen plasma for 5 min. The SEM images of the samples before and after the treatment are shown in Fig. 6(c,d). No significant morphology changes occur after the oxygen plasma treatment. From the PL spectra measured before and after the treatment (cf. Figure 6(e)) we observed that the green emission largely decreases, which is another indication of the correlation between the green emission and the oxygen deficiency.

**Figure 7:**
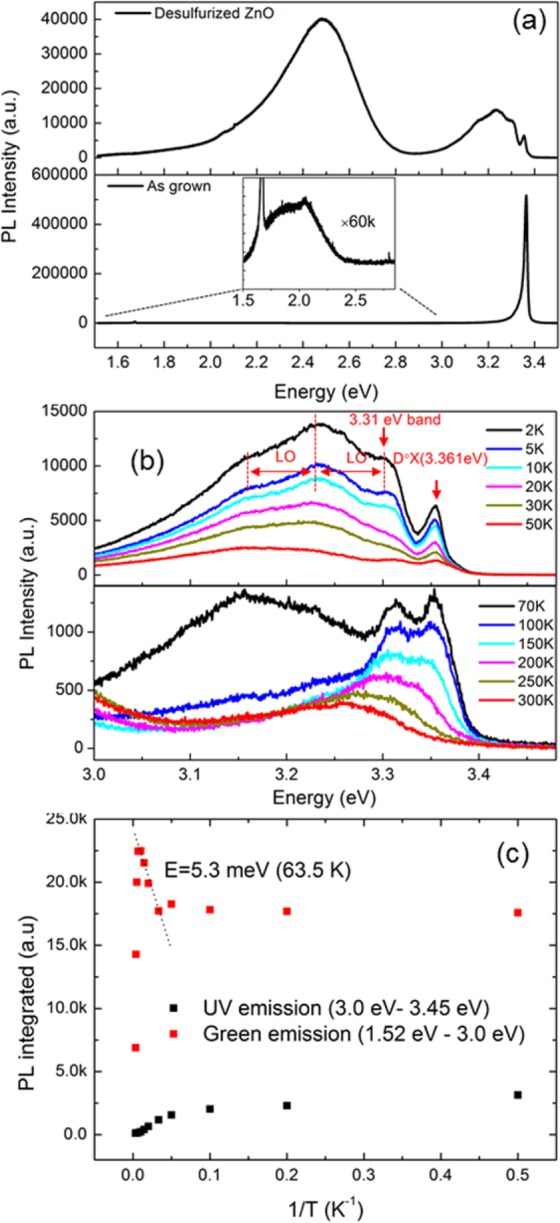
Low temperature PL spectra: (**a**) PL spectra of the as grown and desulfurized ZnO at 2 K are plotted respectively and vertically displaced for clarification. The inset represents the defect emission of the as grown sample (The intensity is increased by 60 times intentionally for clarification). (**b**) UV emission spectra taken from 2 K to 50 K and 70 K to 300 are plotted respectively and vertically displaced for clarification. D°X denotes for the donor bound exciton peak. LO is for the longitudinal-optical phonon energy, which is around 72 meV for ZnO. (**c**) Evolution of the integrated Defect emission and the UV emission as a function of reciprocal temperature. The thermal activation energy of the negative thermal quenching was derived from the linear slope.

To further understand the excitonic behavior of the desulfurized ZnO, the low temperature PL spectra were taken at temperatures ranging from 2 K to 300 K. Figure 7(a) shows the PL spectra of the as-grown and the desulfurized ZnO nanowires taken at 2 K. The as-grown sample shows a single peak at around 3.361 eV, which can be attributed to the neutral donor bound exciton transition (D°X), as reported by Look *et al*.^38^ for bulk ZnO and He *et al*.^39^ for nanorods. The defect emission is shown in the inset figure, which is relatively low as compared with the UV emission. For the desulfurized ZnO, it shows an intense green emission peak. The defect emission to UV emission ratio is much than the as grown one, which suggests that more excitons are trapped by the defects than the neutral donor or free excitons in the desulfurized ZnO.

As we notice that the UV emission of the desulfurized ZnO differs from that of the as grown sample, we plotted the details of the UV emission in Fig. 7(b). The right peak at around 3.36 eV can be assigned to the D°X. Unlike the as grown sample, the dominant excitonic emission peaks are located in the phonon assisted emission range (3.10 eV–3. 31 eV). We note that the three left peaks are equally spaced at 72 meV, which is equal to the longitudinal-optical (LO) phonon energy of ZnO. This leads us to believe that the emission at 3.31 eV (often called A band as well) is the main emission peak, while the other two are its phonon replicas. Several interpretations about the 3.31 eV band have been discussed in the literature, such as first LO phonon replica of free exciton (1LO-FX replica)^40^, stacking fault bound exciton^41^ and surface exciton^42^. For the reports which assign the 3.31 eV to the 1LO-FX replica, it is explained that the electron-phonon coupling in ZnO mainly comes from Frohlich interaction, and such interactions are very small in a perfect crystal due to parity conservation. However, the 1LO-FX replica can be very strong due the presence of surface defects. In a recent work, Tainoff *et al*. reported that both the surface defect states hypothesis and the exciton-phonon interaction are valid to explain the origin of the 3.31 eV band. In our work, we note that the dominant peak at approximately 3.24 eV corresponds to the position of the second phonon replica of FX and the first phonon replica of the 3.31 eV band. Following the discussion in the above reports, we believe that the 3.31 eV band has two accumulated contributions: defect-related transition and 1LO-FX replica. As the desulfurized ZnO is under the oxygen deficiency condition, we can assume that the excitons can be easily trapped by the defects, which will result in a very intense defect–related UV emission. Thus, the dominance of the 3.31 eV band-1LO peak is due to the contributions of both the 1LO of the defect-related transition and the 2LO of the free exciton. We also note that the 3.31 eV band-1LO peak remains dominant until the temperature rises above 70 K. At 100 K, the 3.31 eV band becomes dominant. At this stage, we assign the 3.31 eV band to 1LO-FX replica excluding the contribution of defect-related transition since its phonon replica can no longer be tracked as the temperature rises from 100 K to 300 K.

Figure 7(c) shows another interesting phenomenon of the PL emission, negative thermal quenching (NTQ): wherein the emission intensity increases as the temperature increases. The evolution at each measured temperature shows that the defect emission begins to increase while the temperature rises from 70 K to 150 K, while the UV emission continues to decrease. The NTQ of the green emission has a thermal activation energy of 5.3 meV, which is equal to the thermal energy of the environmental temperature at 63.5 K. This temperature is very close to the beginning temperature of NTQ at around 70 K, which indicates that the NTQ is coming from the defect emission being activated as the temperature increases. The thermally activated excitons can be re-trapped by the defects at the surface. The diffusion length of excitons in ZnO nanowires is relatively large (150–200 nm) at low temperature^43^. Due to the NTQ, the internal quantum efficiency, calculated by taking the ratio between the integrated defect emission and the total emission, is as high as 33%, which is significantly higher than the as-grown sample which is below 1%.

**Figure 8:**
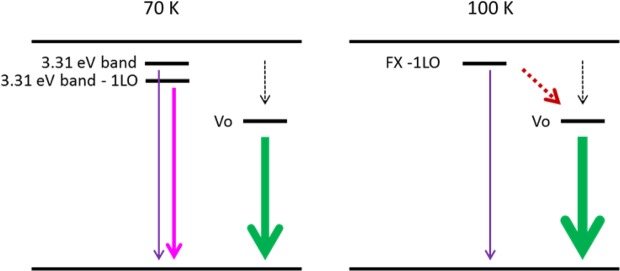
Energy level diagrams summarizing the various mechanisms to explain the dominant 3.31 eV band - 1LO line at 70 K and the negative thermal quenching observed for green emission by proposing the diagram at 100 K. The dotted arrows represent for the nonradiative channels, while the red dotted arrow represents the re-trapping effect of thermally activated exciton by the oxygen vacancies.

By combining the analysis on the thermal evolution of excitonic peaks and the defect emission, we present the energy level diagrams in Fig. 8, which summarizes the various mechanisms to explain the dominance of 3.31 eV band - 1LO peak and the energy transfer of the green emission. At 70 K, the dominant excitonic line is the 3.31 eV band - 1LO peak, which is due to the contributions of the 1LO of defect bound exciton and 2LO of the free exciton peak which were enhanced by the presence of defects generated by the desulfurization process. On the other-hand, photo-excited electrons decay to the oxygen vacancy level via energy transfer channels (dotted arrow), eventually emitting light at 510 nm. When the temperature reaches 100 K, defect bound excitons have been thermally activated, which leads to an additional internal energy transfer channel represented by the red dotted arrow. This channel leads to an increase in the green emitting defect level transition, which corresponds to the NTQ phenomenon. This diagram is strongly supported and aligns well with the results that the 3.31 eV band – 1LO is much weaker at 100 K as compared with 70 K, while the NTQ of the green light occurs with the thermal activation energy corresponding to the ambient thermal energy at around 70 K.

## Conclusion

In summary, desulfurized ZnO was prepared by the chemical deposition method followed by a sulfurization and desulfurization process at a relatively low temperature. The sulfurized ZnO sample has more than 55% ZnS resulting in redshifted bandgap emission and significantly enhanced defect emission. The desulfurization treatment further enhances the integrated defect emission by 42 times as compared with the as grown sample. Our data suggests that the enhancement in green emission comes from a large amount of oxygen vacancies generated during the removal of the sulfur atoms from the sulfurized ZnO. Low temperature PL studies on the desulfurized ZnO sample shows that the phonon-assisted excitonic emission at 3.31 eV - 1LO position can be enhanced by the presence of defects. NTQ was clearly observed from 70 K to 150 K. We propose that by the presence of large amounts of defects, the interaction of excitons with the defects have been enhanced, which induces the dominance of defect bound related emission in the excitonic emission and the strong defect level related transition. The defects can re-trap excitons at higher temperature and the radiative energy relaxation is further enhanced by the re-trapping effect. As for the application, this desulfurization technique has a good potential method served as the technique to synthesize ZnO based green light emitters.
